# Isolated sphenoid sinus opacification is often asymptomatic and is not referred for otolaryngology consultation

**DOI:** 10.1038/s41598-021-89995-7

**Published:** 2021-06-07

**Authors:** Naoki Ashida, Yohei Maeda, Takahiro Kitamura, Masaki Hayama, Takeshi Tsuda, Ayaka Nakatani, Sho Obata, Kazuya Takeda, Hitoshi Akazawa, Fumitaka Inaba, Naohiro Hosomi, Atsuhiko Uno, Hidenori Inohara

**Affiliations:** 1 Department of Otorhinolaryngology–Head and Neck Surgery, Osaka University Graduate School of Medicine, 2-2 Yamada-oka, Suita, Osaka 565-0871 Japan; 2 Department of Otolaryngology-Head and Neck Surgery, Osaka Rosai Hospital, Sakai, Osaka Japan; 3 Department of Otolaryngology-Head and Neck Surgery, Osaka General Medical Center, Osaka, Osaka Japan; 4 Department of Otolaryngology, Yao Municipal Hospital, Yao, Osaka Japan; 5 Department of Diagnostic Imaging, Osaka General Medical Center, Osaka, Osaka Japan; 6 Department of Radiology, Rinku General Medical Center, Izumisano, Osaka Japan

**Keywords:** Diseases, Respiratory tract diseases

## Abstract

Isolated sphenoid sinus opacifications (ISSOs) are clinically important because they can lead to serious complications. However, some patients with ISSOs are asymptomatic, and not all patients are properly referred to the otolaryngology department. Because past studies of ISSOs focused only on patients who received treatment, in this study we selected ISSO cases based on radiology reports, then determined whether these patients had symptoms and were appropriately referred for specialty care. We conducted a retrospective analysis of data collected from patients who underwent computed tomography or magnetic resonance imaging from January 2007 to March 2017 at Osaka General Medical Center. We searched for the terms “sphenoid” or “sphenoidal” using F-REPORT to identify patients who had a sphenoid disease. We checked all selected images and diagnosed ISSOs. Examination of 1115 cases revealed 223 cases of ISSOs, of whom 167 (74.9%) were asymptomatic. We categorized patients with ISSOs into four groups: inflammation, mucocele, fungal diseases, and unclassifiable; the final category was used when edges were irregular or complete opacity was encountered. In the unclassifiable group, the majority of cases required otolaryngology consultation, but 37 of 47 unclassifiable patients did not have an otolaryngology visit. ISSOs are often identified by chance on imaging tests performed by non-otolaryngologists. However, our study revealed that many patients with ISSOs who should be treated by otolaryngologists were not referred to the otolaryngology department. Accordingly, it is important to promote awareness of the disease among other types of clinicians.

## Introduction

Isolated sphenoid sinus opacification (ISSO) is a relatively uncommon disease, but the number of patients diagnosed with ISSOs has increased due to advances in imaging modalities^1^.

The underlying pathologies associated with ISSOs include chronic rhinosinusitis without nasal polyps (CRSsNP), mucoceles, fungal sinusitis, malignant neoplasms, intracranial lesions, benign neoplasms, and chronic rhinosinusitis with nasal polyps (CRSwNP)^2^.

Because ISSOs can lead to serious complications, such as irreversible neurologic defects, intracranial abscess, or meningitis, prompt and thorough management is recommended^3,4^. However, in some ISSOs patients, the early stages are asymptomatic or associated with non-specific symptoms, resulting in a delay of diagnosis.

Some cases of ISSOs may be overlooked, although cases are sometimes identified in the early stages by imaging studies, and may subsequently be referred to the otolaryngology department after the progression of the disease^5^. Past studies of ISSOs have only focused on patients treated by otolaryngologists, and to date, there has been no research on either untreated patients or those not managed by otolaryngologists, or on whether patients are properly referred to an otolaryngology department. Therefore, in this study we identified cases of ISSO based on diagnostic images and investigated whether patients were symptomatic and then referred to an otolaryngology department, diagnosed with ISSO, and received appropriate medical treatment.

In this study, we identified ISSOs cases from a database of imaging studies. We recorded imaging findings, radiographic diagnosis, symptoms, and how each ISSOs patient was managed. We referred to diagnostic reports by radiologists and reviewed all images that were identified by keywords. Past studies that only focused on treated cases reported that the majority of patients were symptomatic. By contrast, we found that the majority of ISSOs patients were asymptomatic, and therefore may not have been properly referred to otolaryngology. Consequently, as noted above, the results suggest that analysis limited to treated patients may be biased. In this study, we revealed a new aspect of ISSOs by identifying cases based on imaging results.

## Material and methods

We conducted a retrospective analysis of data collected from patients who underwent CT or MRI from January 2007 to March 2017 at Osaka General Medical Center. During the study period, the images were read by physicians who subspecialized in head and neck imaging. We searched for the terms “sphenoid” or “sphenoidal” using F-REPORT to select patients who had a sphenoid disease. We checked all selected images and diagnosed ISSOs. We performed a retrospective chart review to determine whether patients with ISSOs were appropriately referred to an otolaryngology department. This study was approved by the institutional review board of Osaka General Medical Center (#OGMC2021-017).

Our primary study inclusion criterion was ISSO involving only the unilateral sphenoid sinus. Patients were excluded if they had ISSOs involving the bilateral sphenoid sinuses or other sinuses, prior skull base surgery or transnasal pituitary surgery, brain tumor (including suspected cases), disease of the sphenoid bone (including suspected cases), or acute head trauma.

In each case, we evaluated the following: mucosal thickness; the presence or absence of an air-fluid level and air bubbles; and whether the lesion was round or expansive, completely opaque, irregularly shaped, and showed calcification on CT and/or the flow void sign on MRI.

Based on imaging, we categorized the cases as follows: inflammation, fungal disease, mucocele, and unclassifiable. Inflammation was diagnosed based on mucosal thickness, air-fluid level formation, and the presence of air bubbles. Fungal disease was diagnosed based on high-density areas or calcification on CT, or low-intensity areas on T2-weighted MRI images. Mucocele was diagnosed based on expanding lesions. Unclassifiable cases were those with complete opacity or in which the border was not smooth.

We also checked medical records to confirm that patients with ISSOs were treated properly, including the following details: recording of ISSOs, consultation with the otolaryngology department, and treatment of ISSOs.

Finally, we reviewed the patient’s symptoms in the medical record: headache, dizziness, etc.

### Ethics approval

This study was approved by the institutional review board of Osaka General Medical Center (#OGMC2021-017).

## Results

**Figure 1:**
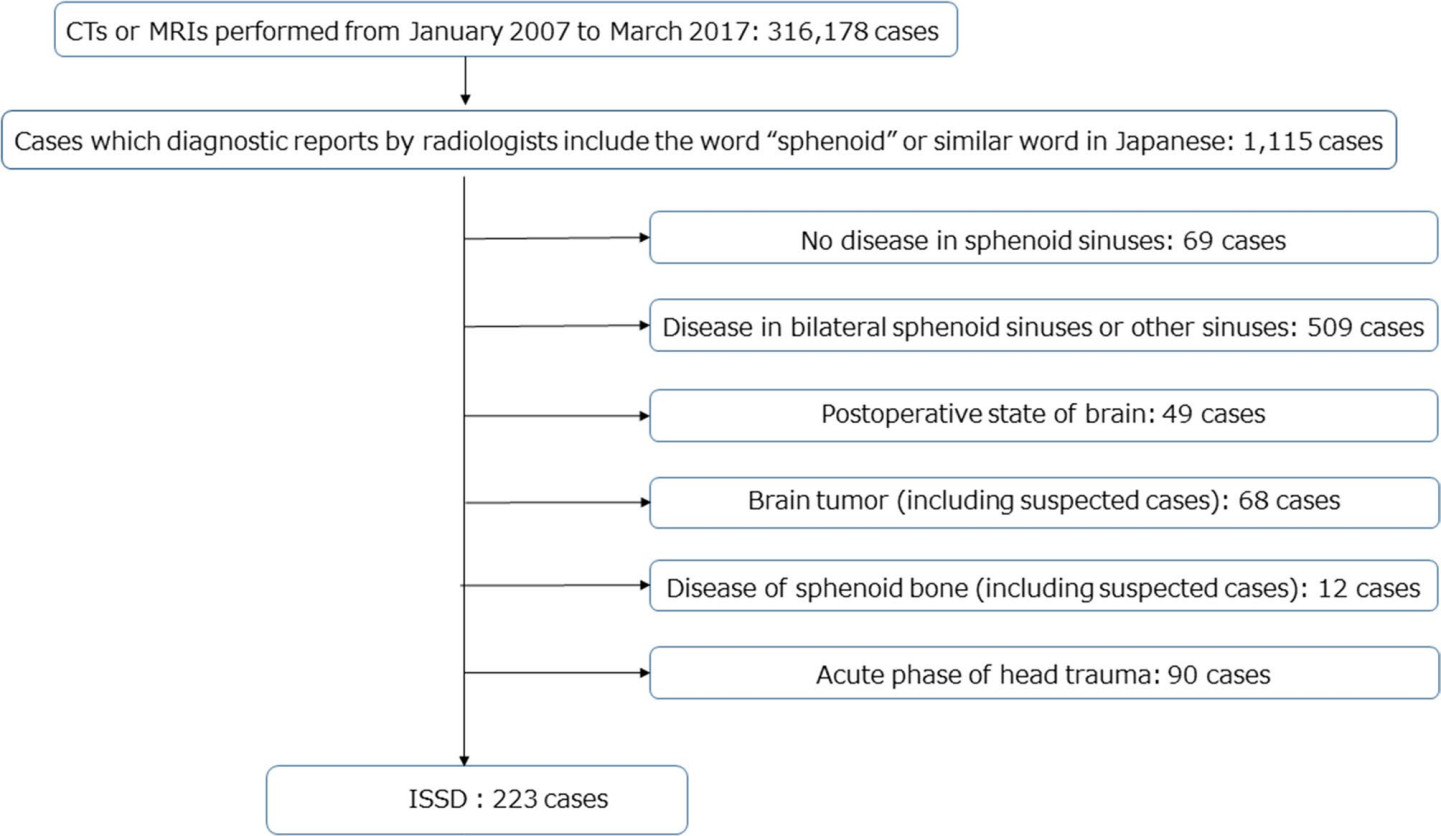
Patient selection diagram.

CT and MRI were performed in 316,178 cases. All cases were checked and reported by radiologists; 1115 cases included the terms “sphenoid,” “sphenoidal,” or the equivalent expressions in Japanese. We checked all 1115 cases and excluded the cases described above, e.g., 68 cases of brain tumor and 12 cases involving the sphenoid bone. Ultimately, 223 cases met the criteria (Fig. 1).

The clinical backgrounds of the 223 patients with ISSOs are shown in Table 1. Twenty-two patients were examined by otolaryngology, and 201 patients were examined by other departments. The results for all cases are shown in Table 2. The diagnoses are shown in Table 3.

**Table 1:** The background of ISSOs patients.

Cases	223
**Age**	
Median	69
Range	6–93
**Sex**	
Male	128
Female	95
**Modality**	
CT alone	74
MRI alone	101
**CT and MRI**	48
Brain MRI	139
Head CT	81
Sinus CT	24
Sinus MRI	4
Other CTs	17
Other MRIs	6

**Table 2:** Imaging findings of ISSOs patients.

Mucosal thickening	83 (37.2%)
Niveau formation	56 (25.1%)
Air bubbles	22 (9.8%)
Round lesion	23 (10.3%)
Expanding lesion	6 (2.6%)
Complete opacity	74 (33.2%)
Irregular shape	6 (2.7%)
Calcification in CT	16 (7.2%)
High density area in CT	4 (1.8%)
Flow void in T2-weighted MRI	25 (11.2%)

**Table 3:** Diagnosis groups by imaging.

Inflammation	116 (52.0%)
Mucocele	28 (12.6%)
Fungal disease	32 (14.3%)
Unclassifiable	47 (21.1%)

In cases of ISSOs, 48 patients were examined by otolaryngologists, and 172 patients were not (Table 4). Diagnoses were as follows: inflammation in 93 cases (54.0%), mucocele in 18 cases (10.5%), and fungal disease in 24 cases (14.0%); 37 cases were unclassifiable (21.5%).

**Table 4:** Otolaryngology consultation.

Yes	48
**No**	
Inflammation	93 (54.0%)
Mucocele	18 (10.5%)
Fungal disease	24 (14.0%)
Other diseases	37 (21.5%)
Unknown	3

In regard to symptoms, 167 patients (74.9%) were asymptomatic and 56 patients were symptomatic (Table 5). Of those, 22 patients had headaches and detectable sphenoid sinus disease. Ten of those patients were not consulted about ISSOs. Other symptoms included dizziness (12 patients; 5.4%), face/eye pain (4 patients; 1.8%), postnasal drip (2 patients; 0.9%), diplopia (2 patients; 0.9%), and other symptoms (16 patients; 7.2%).

**Table 5:** Symptoms of ISSOs patients.

Asymptomatic	167 (74.9%)
**Symptomatic**	
Headache	22 (9.9%)
Dizziness	12 (5.4%)
Face/eye pain	4 (1.8%)
Post nasal drip	2 (0.9%)
Diplopia	2 (0.9%)
Others	16 (7.2%)

## Discussion

ISSOs are defined as unilateral sphenoid sinus disease with no disease in other sinuses. ISSOs encompasses multiple conditions, including inflammation, fungal disease, and tumors. In some cases, ISSOs can lead to serious intracranial and orbital complications^2^. 

In recent years, there has been an increase in the number of opportunities for imaging studies, potentially leading to an increase in the number of incidental ISSOs findings^6^. In cases with sinus disease, the morbidity of ISSOs is 2.7%^7^. 

In ISSOs, the most common diagnosis is inflammation, including rhinosinusitis (bacterial infection), mucocele, and fungal disease. In previous research, the frequency of inflammation was reported as 38–65%^1,2,8,9^. In this study, the total percentage of cases with inflammation, mucocele, and fungal disease was 81%. This may be because this study included many cases of mild inflammation that are incidentally observed in imaging studies, whereas previous reports have examined cases of treatment.

The frequency of benign and malignant neoplasms in ISSOs is 6–19%^1,2,8,10^. In this study, the “unclassifiable” category included benign and malignant neoplasms. The percentage of cases considered to be unclassifiable was lower than the frequency of benign and malignant neoplasms in previous studies because we excluded diseases that extended outside the sphenoid sinus.

ISSOs can lead to a variety of symptoms, with headache being the most common: 62–88% of ISSOs cases are associated with headaches^1,11–13^. Regions and types of headaches are not specific to ISSOs^6,12^. In this study, 75% of patients had no symptoms. The reason for this is that previous studies focused on analyzing patients who were operated on, so more of those patients were symptomatic. On the other hand, previous studies may have overestimated the frequency of symptoms because they examined only patients who were surgically treated. Other symptoms, such as nasal obstruction, rhinorrhea, and eye or facial pain, were observed, but none of these symptoms are specific. Sieskiewicz et al. reported that among 32 ISSOs cases, nine had complications such as low vision and meningitis^5^. We should consider the possibility of ISSOs in patients with headache.

Our particular focus was on headache, a symptom that is often seen in departments other than otolaryngology. Of the patients reviewed in this study, 22 had ISSOs with a primary complaint of headache, and ten of these cases had not been referred to otolaryngology.

Friedman et al. reported that 34% of ISSOs patients look normal in endoscopy, suggesting that imaging tests are necessary to diagnose sphenoid diseases^1^. CT is effective for detecting bone erosion, and MRI for qualitative evaluation. Sensitivities of CT and MRI in diagnosing inflammatory lesions are 95% and 61%, respectively, whereas, in tumorous disease, the corresponding values are 72% and 100%, respectively. In the osseous disease, sensitivity is 100% for both CT and MRI, whereas, in sphenoid sinus roof defect, the sensitivities are 50% and 100%, respectively^6^. These data suggest that ISSOs can be diagnosed with a certain degree of accuracy by CT and MRI. Thus, although our data have not been validated by surgery or pathology, it seems reasonable to assume that they are reliable.

In this study, we classified the diagnosis from imaging alone in all cases. In order to improve the diagnosis rate, we included the category “unclassifiable case,” which means that the case should be referred to specialists. Because the frequency of the disease did not differ significantly from those reported in previous studies, we believe that the diagnoses in this study were reliable.

Several studies of incident sinus lesions found on imaging studies have been published in the past. For example, Lloyd et al. reported that CT exhibited sinus abnormalities in 39% of asymptomatic adults^14^. Cooke et al. reported that 37.5% of adults had incidental abnormalities on MRI^15^. Currently, there is no consensus view about how to treat asymptomatic sinusitis. However, it is necessary to pay attention to patients who have lesions in the sphenoid sinus even if they are asymptomatic, because the sphenoid sinus is adjacent to many important structures. Many ISSOs found in this study seemed to be asymptomatic and were therefore left untreated, primarily due to the location and size of the lesion. In other cases, ISSOs were recognized but were untreated, suggesting that many physicians underestimate the risks of sphenoid sinus disease. Treatment is not necessary for cases in which the lesion is apparently mild, e.g., mild mucosal thickening or a lesion suspected of a small mucocele. However, various symptoms can appear upon exacerbation, and it is necessary to provide a sufficient explanation and follow-up of symptoms. If a sphenoidal sinus lesion is found by chance on imaging, a closer examination by an otolaryngologist is recommended in two cases: when neoplastic or fungal diseases cannot be ruled out, and when symptoms of sphenoidal sinus lesions such as headache or ocular symptoms cannot be ruled out. In this study, however, ISSOs whose primary symptom was headache was identified in 22 patients, of whom 10 had not been referred to otolaryngology. In addition, the patients we classified as “unclassifiable” should have seen an otolaryngologist because they could not be ruled out as tumors on imaging, but only 5 of 47 “unclassifiable” patients were referred to otolaryngologists (data not shown). All doctors, not only otolaryngologists, should have sufficient knowledge of sphenoid sinus lesions.

Our study of ISSOs is the first that is not limited to treated cases, although it has some limitations. The most important of these is that the results were not confirmed pathologically. For example, in our study, mucoceles with complete sphenoid opacification could be classified into the “unclassifiable” category. However, these patients should be referred to otolaryngologists, and therefore the number of patients that should be referred to specialists is correct in this context. In addition, it is also possible that the patient was instructed to see an otolaryngologist but this was not indicated in the medical records due to incomplete entries.

However, our results suggest that even when non-treated cases are considered, ISSOs comprise a group of diseases similar to those reported in the past. The number of patients without symptoms was considerably higher than previously reported. We also believe that many cases were not properly referred for specialty care because their importance was underestimated by physicians in departments other than otolaryngology. In recent years, opportunities for head imaging have increased; accordingly, there is a need to educate the public about ISSOs.

## Conclusion

We reviewed 223 cases of ISSOs. Previous reports indicated that 62–88% of cases have headaches, but our findings revealed that 74.9% of cases were asymptomatic. Of the 47 cases that should have been referred to otolaryngology, 37 were not, suggesting the need for more widespread awareness of ISSOs.
